# ADHD symptom profiles, intermittent explosive disorder, adverse childhood experiences, and internalizing/externalizing problems in young offenders

**DOI:** 10.1007/s00406-020-01181-4

**Published:** 2020-08-11

**Authors:** Steffen Barra, Daniel Turner, Marcus Müller, Priscilla Gregorio Hertz, Petra Retz-Junginger, Oliver Tüscher, Michael Huss, Wolfgang Retz

**Affiliations:** 1 Institute for Forensic Psychology and Psychiatry, Saarland University Hospital, Kirrberger Str. 100, 66421 Homburg, Germany; 2 Department of Psychiatry and Psychotherapy, University Medical Center of the Johannes Gutenberg-University, Mainz, Germany; 3 Department of Child and Adolescent Psychiatry, University Medical Center of the Johannes Gutenberg-University, Mainz, Germany

**Keywords:** Attention deficit hyperactivity disorder, Childhood trauma, Crime, Psychopathology, Juvenile justice

## Abstract

Attention-deficit/hyperactivity disorder (ADHD) and co-existing psychiatric/psychological impairments as well as adverse childhood experiences (ACEs) are common among young offenders. Research on their associations is of major importance for early intervention and crime prevention. Intermittent explosive disorder (IED) warrants specific consideration in this regard. To gain sophisticated insights into the occurrence and associations of ADHD, IED, ACEs, and further psychiatric/psychological impairments in young (male and female) offenders, we used latent profile analysis (LPA) to empirically derive subtypes among 156 young offenders who were at an early stage of crime development based on their self-reported ADHD symptoms, and combined those with the presence of IED. We found four distinct ADHD subtypes that differed rather quantitatively than qualitatively (very low, low, moderate, and severe symptomatology). Additional IED, ACEs, and further internalizing and externalizing problems were found most frequently in the severe ADHD subtype. Furthermore, females were over-represented in the severe ADHD subtype. Finally, ACEs predicted high ADHD symptomatology with co-existing IED, but not without IED. Because ACEs were positively associated with the occurrence of ADHD/IED and ADHD is one important risk factor for on-going criminal behaviors, our findings highlight the need for early identification of ACEs and ADHD/IED in young offenders to identify those adolescents who are at increased risk for long-lasting criminal careers. Furthermore, they contribute to the debate about how to best conceptualize ADHD regarding further emotional and behavioral disturbances.

## Introduction

Delinquency committed by adolescents and young adults is a common phenomenon; however, whereas most juveniles overcome offending when entering adulthood, some of them continue to develop long-lasting criminal careers [1]. With respect to their future perspectives, the economic costs, and the safety of our society, it is essential to identify young offenders who are at risk of continuous crime at an early stage of their criminal development.

Psychiatric impairments have been related to elevated risk of delinquency in young people: High rates of various psychiatric disorders were found among young detainees [2–5]. Although internalizing problems must not be neglected, externalizing problems are usually more prevalent [2]. Moreover, young offenders with high expressions of externalizing behavior problems carry an elevated risk of criminal recidivism compared to young people without or with low expressions of externalizing symptomatology [5–8].

Attention-deficit/hyperactivity disorder (ADHD) is one externalizing disorder, which has received increased attention in research on juvenile and adult delinquency. Previous findings indicate that children with ADHD (with and without psychiatric comorbidity) show an elevated risk of early, persistent, and versatile crime involvement [9–13]. Meta-analyses point to ADHD prevalence rates of 26–30% within juvenile and adult detention samples, reflecting a five- to tenfold risk in comparison to the general population [14, 15]. Young detainees with ADHD showed faster and higher reoffending rates than those without ADHD [16]. Moreover, offenders with ADHD tend to be more frequently involved in impulsive-reactive violent activities than in proactive-premeditated criminality [17].

However, research on the association between ADHD and delinquency has faced several complications. There has been an ample debate about the conceptualization of ADHD. Sole reliance on an ADHD diagnosis risks to undermine specific differences among the three presentations (predominantly inattentive, hyperactive-impulsive, or combined) described in the Diagnostic and Statistical Manual of Mental Disorders (DSM-5) [18]; yet, the empirical distinctiveness of these presentations has also been criticized [19–21]. Moreover, ADHD is often accompanied by further emotional and behavioral problems that are not represented by the given diagnostic criteria. There is an ongoing scientific debate whether such symptoms display characteristics of comorbid psychiatric disorders or constitute to the core symptomatology of ADHD [22–27]. Yet, the consideration of these symptoms is imperative when exploring the associations of ADHD with delinquency as they might affect further crime development [28, 29].

With regard to further examining emotional and behavioral problems accompanying ADHD, one psychiatric disorder appears of specific relevance: intermittent explosive disorder (IED). According to DSM-5 [18], IED reflects repeated acts of impulsive-aggressive outbursts (verbal or physical, against humans, animals or objects), which are clearly disproportionate to the given situation. Considering the simultaneous occurrence of ADHD and IED in young offenders is necessary taking into account the similarities of both disorders on a behavioral level and on underlying processes as well as their association with criminal behavior [23, 30, 31]. IED ranged among the most commonly reported disorders among adolescents in the US National Comorbidity Survey (14.1%), and both ADHD and IED were predictive for reported crime [32]. IED rates of 5–11% were found in juvenile and adult offender samples [33, 34]. DeLisi et al. [35] highlighted that “by its very definition, IED is an important clinical disorder with explicit linkages to criminal offending; however, the construct has been largely overlooked by researchers”. They found IED to be predictive of violent offending and persistent crime involvement and recommended to further investigate the distinct occurrence of IED in offender samples and not only consider respective symptomatology as affiliated to other behavioral disorders such as ADHD.

Additionally, neglecting shared etiological factors that contribute to ADHD, co-existing emotional/behavioral problems, and delinquency holds the risk to draw inadequate conclusions and implications. Adverse childhood experiences (ACEs) display such influencing factors. ACEs are common among young offenders and were proven to contribute to ADHD and the occurrence and maintenance of delinquency [3, 36–44]. ACEs were also associated with IED over and above their effects on other psychiatric disorders [45–47]. Research has proposed that the association of ACEs, ADHD, and IED with aggressive behavior may rely on distorted social cognition processes as a consequence of, e.g., dysfunctional social learning experiences [39, 48–50].

The present study intended to overcome some of the abovementioned shortcomings of previous research (e.g., categorical consideration of ADHD diagnosis or the neglect of co-occurring emotional/behavioral problems and shared risk factors such as ACEs). First, according to the dimensional character and heterogeneity of ADHD [16, 23, 51], we used latent profile analysis (LPA) to empirically derive mutually exclusive, homogeneous subtypes of ADHD in a juvenile detention sample. Similar approaches have been successfully used to examine the heterogeneity of (a) ADHD in non-forensic samples [52], and (b) adolescent delinquents regarding other disruptive behavior problems like oppositional defiant disorder [8] and conduct disorder [7], as well as ACEs [53]. However, to the best of our knowledge, no study has yet followed a latent class/profile approach to investigate subtypes of ADHD among young offenders. Second, we aimed at extending previous research by focusing on IED as a relevant representation of emotional/behavior problems co-occurring with ADHD. Since both ADHD and IED have been shown to co-exist with further psychiatric impairments [54–58], we also considered further internalizing and externalizing problems. Moreover, we accounted for the effects of ACEs in the associations of ADHD, IED and further internalizing and externalizing problems within young offenders. Furthermore, because our sample consisted of male and female offenders, we were able to investigate potential sex differences. Research on the associations of ADHD and crime in female offenders is rare and existing studies have so far yielded inconclusive results [4, 59]. Based on previous research, we expected to detect at least two subtypes of young offenders reflecting high and low expressions of ADHD. We anticipated higher rates of IED compared to general population samples, especially in co-occurrence with elevated ADHD severity. We assumed that participants high on ADHD and/or IED had increased rates of ACEs and showed further internalizing and externalizing problems more frequently. Due to the scarcity of research on ADHD in female offenders, we examined sex differences in exploratory manner.

## Methods

### Procedure

The present study was conducted as part of a pilot study that aimed to examine the feasibility of scientific data assessment in the below-mentioned juvenile detention center. The study protocol was approved by the ethical review board of the Medical Council in Rhineland-Palatine, Germany (reference number: 837.290.17 (11124); approval date: 21st September 2017). Study procedures were performed in accordance with the ethical standards of the Declaration of Helsinki. Data assessment took place at the juvenile detention center in Worms, Germany. The German juvenile law is usually applied for young people aged 14–21 years (at the time of offending); however, the maximum age may be expanded under specific circumstances (e.g., when several offences committed at different points in time (age periods) are summarized for one verdict). Juvenile detention is defined as an educational intervention for adolescents and young adults. In contrast, youth prison reflects a measure of punishment. Thus, juvenile detention is usually arranged for adolescents or young adults with rather minor offenses who have not yet been involved in chronic crime. The maximum duration of juvenile detention is 30 days. All adolescents and young adults who had to stay for at least 7 days in juvenile detention between May 2018 and May 2019 were provided study information via mail about three weeks before the beginning of sentence. There were no further inclusion or exclusion criteria. The document contained information about (1) study procedures, (2) the voluntariness of participation including the possibility to withdraw consent at any time, (3) the scientific purpose of data collection, (4) the anonymization of data, and (5) the fact that consent or denial of study participation would not have any consequences concerning the given sentence.

In case of study participation, written informed consent was given by the detainee (and his/her legal guardians when aged below 18 years) at the beginning of sentence. Self-report questionnaires were handed over on the first day of detention to be filled out at the detainees’ private detention rooms. Completing the questionnaires took about one hour. In case a participant did not understand the content of single items, she/he could ask a responsible staff member at the juvenile detention center for assistance. Completed questionnaires were collected in a closed box.

### Participants

Since the present study represents (parts of) a pilot/feasibility study, we did not apply any sample size calculations in advance. Data were collected from a consecutive sample of a total of 161 adolescents or young adults (134 males, 27 females) with a mean age of 18.48 years (SD = 2.1; range = 14–25 years). For the present study, data were considered only from those detainees who had given full information on the questionnaires concerning ADHD and IED symptoms, leaving a total of 156 participants (129 male, 82.7%; 27 female, 17.3%) between 14 and 25 years (*M* = 18.53 years, SD = 2.13 years). The mean length of detention was 2.11 weeks (SD = 0.68 weeks, range = 1–4 weeks). Detainees showed an average school education of 9.29 years (SD = 0.75 years, range = 8–13 years). Males and females did not differ concerning age, length of detention, or years of education (*p* > 0.05).

Index offenses (most severe per participant) included (grievous) bodily harm (*n* = 34; 21.8%), property offenses (*n* = 56; 35.9%), breach of narcotics law (*n* = 26; 16.7%), breach of school law/excessive school skipping (*n* = *20*; 12.8%), driving without driver`s license (*n* = 9; 5.8%), and others (*n* = 11; 7.1%). No sex differences were found, *χ*^2^(5) = 4.46, *p* = 0.485.

### Questionnaires

#### Self-report Wender-Reimherr adult attention deficit disorder scale (SR-WRAADDS)

ADHD symptoms based on the Utah Criteria were assessed by the German version of the SR-WRAADDS (WR-SB) [51, 60, 61], which comprises 59 items evaluated on a five-point Likert-scale (1 = not at all to 5 = very much). Items were summed up to 10 scales: (1) attention difficulties, (2) hyperactivity/restlessness, (3) temper, (4) affective lability, (5) emotional over-reactivity, (6) disorganization, (7) impulsivity, (8) oppositional symptoms, (9) academic problems, and (10) social attitude.

Satisfactory psychometric properties were proven for the English and German versions of the WR-SB [51, 60]. In the present study, Cronbach’s *α* for all subscales were between 0.77 and 0.90.

#### Intermittent explosive disorder-screening questionnaire for DSM-5 (IED-SQ)

IED was assessed using the IED-SQ [62]. The IED-SQ consists of two parts. Part 1 contains five items relating to the frequency of aggressive behaviors on a six-point Likert-scale (0 = never happened to 5 = happened “so many” times that I cannot give a number) that can be summed up to an IED total aggression score. Part 2 contains five items asking for additional aggression-related behaviors, namely (1) weekly arguments/temper outbursts, (2) annual number of aggression against people or property, (3) planned or unplanned aggression, (4) concern about/problems because of aggression, and (5) aggression without the influence of any substances. Indication of DSM-5 IED is based on a combination of an IED total aggression score of at least 12 and the fulfillment of part 2 items according to given scoring criteria (please consider to the given references for detailed scoring information). For the present study, the IED-SQ was translated into German by the shared first author and independently back-translated into English by two German master’s degree psychologists who were fluent in English and blind to the original IED-SQ. Back-translations were compared to the original IED-SQ by an English native speaker.

Good psychometric properties were proven for the IED-SQ, e.g., regarding the accordance with clinical diagnoses (*к* = 0.80), test–retest reliability (*к* = 0.71), 82% sensitivity, 97% specificity, and overall accuracy = 0.90 [62]. In the present study, internal consistency of the IED total aggression score was good (Cronbach’s *α* = 0.79).

#### Childhood trauma questionnaire-short form (CTQ-SF)

The German version of the 28-item CTQ-SF [63, 64] was used to assess ACEs in form of emotional abuse, emotional neglect, physical abuse, physical neglect, and sexual abuse occurred between the ages of 0 to 18 years. Items were scored on a 5-point Likert-scale from 0 (= never true) to 4 (= very often true). A CTQ-poly score was calculated to represent poly-victimization following a procedure applied in previous studies [42, 65]. First, items rated as 0 and 1 were coded as not present and items rated as 2 to 4 were coded as present. Second, CTQ-subscales were rated as fulfilled if one respective item was coded as present. Third, a CTQ-poly score was built by summing up the number of fulfilled subscales.

The English and German CTQ-(SF) showed good psychometric properties [63, 64, 66]. In the present study, internal consistencies for the subscales were between Cronbach’s *α* = 0.64 (physical neglect) and *α* = 0.96 (emotional neglect).

#### Youth self-report (YSR)

Perceived impairments during the last six months were assessed using the 103-item YSR [67, 68]. The occurrence of each item is rated as 0 (= never), 1 (= sometimes), or 2 (= always). Items can be assigned to eight subscales, which build up to three problem scales: (1) internalizing problems (withdrawn/depressed, somatic complaints, anxious/depressed; (2) externalizing problems (rule-breaking behavior, aggressive behavior); and (3) mixed problems (thought problems, attention problems, social problems). Clinical significance is present when scale scores exceed certain *T*-values provided in the manual. For the present study, internalizing and externalizing problem scales were considered.

The German version of the YSR showed satisfactory psychometric properties [68]. Internal consistency was good in the present study (internalizing problems: Cronbach’s *α* = 0.77; externalizing problems: Cronbach’s *α* = 0.87).

### Statistical analyses

Analyses were conducted in IBM SPSS version 26.0 for Windows and in R (R Core Team, 2020). Person-centered ADHD subtypes were empirically derived by latent profile analysis (LPA) using the tidyLPA package in R [69]. The ten WR-SB scale scores served as indicators. The best fitting model was selected under consideration of several fit indices (see below). Participants were assigned to latent profiles according to their highest affiliation probability based on maximum likelihood estimations. To compare ADHD subtypes regarding further variables, we performed parametric and non-parametric analyses, e.g., *χ*^2^-statistics, ANOVAs, and MANOVAs with post-hoc Bonferroni or Games–Howell tests as well as linear and multinomial logistic regressions. Results were considered as statistically significant with *p*-values below 0.05.

## Results

### LPA on ADHD symptomatology

**Fig. 1:**
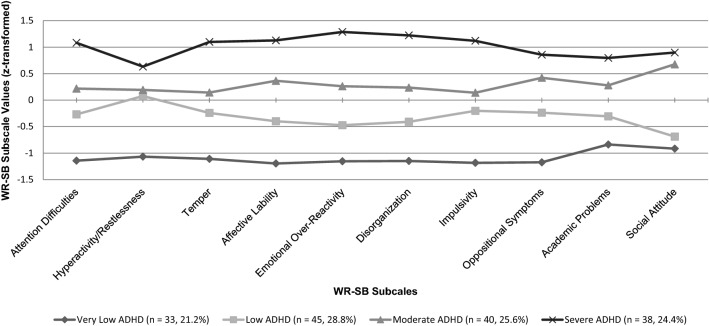
LPA subtypes based on WR-SB subscale values (*z*-transformed)

Models with one to ten profiles were compared using a hierarchical analytical process provided by the tidyLPA command in R [70], which includes several fit indices, e.g., the Akaike Information Criterion [71], Approximate Weight of Evidence Criterion [72], Bayesian Information Criterion [73], Classification Likelihood Criterion [74], and Kullback Information Criterion [75]. The model with four latent profiles fitted our data best. The entropy value of 0.93 indicated clear assignments of participants to latent ADHD profiles [76]. Profiles are presented in Fig. 1 (based on standardized *z*-values). According to their differences on overall ADHD severity, we labeled them (1) very low, (2) low, (3) moderate, and (4) severe ADHD subtypes.

### Descriptive differences among subtypes

Descriptive data of LPA derived subtypes are presented in Table 1. Male participants were over-represented in the low ADHD subtype, females were over-represented in the severe subtype. Participants of the severe subtype were significantly older than those of the low ADHD subtype. No differences emerged regarding index offenses, length of detention, or years of education.

**Table 1:** Descriptive differences among ADHD subtypes

	ADHD subtypes				
	Very low (*n* = 33)	Low (*n* = 45)	Moderate (*n* = 40)	Severe (*n* = 38)	
	*n* (AR)	*n* (AR)	*n* (AR)	*n* (AR)	*χ*^2^ (*df*)
Sex					
Male	28 (0.4)	45 (3.6)	31 (− 1.0)	25 (− 3.2)	17.86 (3)***
Female	5 (− 0.4)	0 (− 3.6)	9 (1.0)	13 (3.2)	
Index offenses					
Bodily harm	8 (0.4)	8 (− 0.8)	11 (1.0)	7 (− 0.6)	12.83 (15)
Property offenses	15 (1.3)	17 (0.3)	5 (− 0.8)	14 (0.1)	
Breach of narcotics law	5 (− 0.3)	8 (0.2)	5 (− 0.6)	8 (0.8)	
Beach of school law/excessive school skipping	2 (− 1.3)	6 (0.1)	7 (1.0)	5 (0.1)	
Driving without driver’s license	1 − 0.8)	5 (1.8)	2 (− 0.2)	1 (− 1.0)	
Others	2 (− 0.3)	6 (0.1)	7 (1.0)	5 (0.1)	
IED diagnosis	1 (− 4.4)	10 (− 2.3)	16 (0.6)	29 (6.0)	46.42 (3)***
YSR (categorical)					
Internalizing problems	1 (− 2.7)	1 (− 3.5)	15 (3.2)	14 (3.0)	29.36 (3)***
Externalizing problems	0 (− 2.6)	2 (− 2.2)	10 (2.3)	10 (2.5)	24.05 (3)***

	*M* (SD)	*M* (SD)	*M* (SD)	*M* (SD)	*F* (3, 146)
Age	18.29 (2.41)_a, b_	18.22 (1.39)_a_	18.23 (2.22)_a,b_	18.55 (2.11)_b_	3.34*
Length of detention (weeks)	2.23 (0.72)	2.10 (0.66)	2.10 (0.71)	2.03 (0.68)	0.50
Years of education	9.36 (0.98)	9.42 (0.63)	9.13 (0.61)	9.26 (0.83)	1.08
WR-SB					
Attention difficulties	10.26 (3.12)_a_	14.63 (3.22)_b_	17.03 (3.48)_c_	21.45 (3.29)_d_	69.92***
Hyperactivity/restlessness	4.90 (1.42)_a_	8.71 (3.27)_b_	8.98 (2.42)_b_	10.39 (2.83)_b_	26.89***
Temper	4.29 (1.04)_a_	7.39 (2.87)_b_	8.62 (2.44)_b_	11.95 (2.17)_c_	66.04***
Affective lability	6.58 (1.89)_a_	9.54 (2.41)_b_	12.48 (1.96)_c_	15.34 (2.13)_d_	110.30***
Emotional over-reactivity	5.74 (0.93)_a_	8.17 (1.79)_b_	11.03 (2.08)_c_	14.84 (2.05)_d_	166.04***
Disorganization	8.00 (1.53)_a_	12.46 (3.65)_b_	16..18 (3.27)_c_	22.00 (3.69)_d_	118.14***
Impulsivity	7.87 (1.69)_a_	12.66 (3.10)_b_	14.20 (3.52)_b_	18.79 (2.91)_c_	80.31***
Oppositional symptoms	14.97 (4.94)_a_	22.15 (4.57)_b_	26.48 (4.53)_c_	29.53 (5.08)_d_	59.50***
Academic problems	5.10 (1.66)_a_	6.90 (2.64)_b_	8.75 (2.71)_c_	10.47 (3.47)_c_	25.15***
Social attitude	14.52 (4.56)_a_	15.68 (3.88)_a_	25.50 (4.26)_b_	27.06 (4.67)_b_	82.82***
IED total aggression score	10.13 (4.54)_a_	11.29 (5.10)_a, b_	13.43 (5.56)_b, c_	16.13 (5.33)_b, c_	9.35***
CTQ poly score	2.03 (1.17)_a, b_	2.00 (1.40)_a_	2.63 (1.51)_a, b_	2.89 (1.43)_b_	3.78*
YSR (dimensional)					
Internalizing problems	3.97 (4.90)_a_	4.44 (5.69)_a_	13.98 (11.01)_b_	13.84 (13.31)_b_	12.75***
Externalizing problems	6.07 (7.21)_a_	7.22 (7.39)_a_	13.40 (11.16)_b_	15.76 (12.26)_b_	8.34***

*AR* adjusted residuals. Significant deviations from expected distribution with AR ≤ − 2.0 or AR ≥ 2.0. Groups with the same subscripts (a, b, c, d) did not significantly differ from each other (*p* ≥ 0.05). *p < 0.05, ***p ≤ 0.001.

Subtypes differed significantly among each other in ascending order (very low, low, moderate, severe ADHD) on the WR-SB scales attention difficulties, affective lability, emotional over-reactivity, disorganization, and oppositional symptoms. Significant overall differences were also found on all other WR-SB subscales showing increasing values with elevated ADHD severity; yet, not all subtypes differed significantly from each other.

IED total aggression scores increased with elevated ADHD severity. The very low ADHD subtype differed significantly from the moderate and severe subtype, and the low subtype differed significantly from the severe subtype. A total of 56 participants (35.90%) fulfilled the criteria for DSM-5 IED diagnosis. Participants with IED diagnosis were over-represented in the severe ADHD subtype and under-represented in the low and very low subtypes.

Regarding the CTQ-SF, 85.3% of the total sample reported at least one ACE. More specifically, 18 participants (11.5%) reported one ACE, 31 (19.9%) reported two ACEs, 40 (25.6%) reported three ACEs, 41 (26.3%) reported four ACEs, and 3 (1.9%) reported five ACEs. The mean CTQ-poly score was 2.43 (SD = 1.42). No sex differences were found (*p* = 0.212). ADHD subtypes differed significantly on CTQ-poly scores. The severe ADHD subtype showed the highest score with significantly more ACEs than the low subtype.

According to the YSR, 19.9% (*n* = 31) and 32.1% (*n* = 50) of the total sample showed clinically significant scores on internalizing and externalizing problems, respectively. No sex differences emerged for internalizing problems, but females were over-represented among those with clinically significant externalizing problems (*n* = 14, 51.9%, AR = 2.4), *χ*^2^(1) = 5.88, *p* = 0.015. Participants from the moderate and severe ADHD subtypes showed clinically significant internalizing and externalizing problems more frequently than other participants. ADHD subtype differences were also found on dimensional internalizing and externalizing problem scores. Scores of the very low and low ADHD subtypes were significantly lower than scores of the moderate and severe subtypes.

### Associations among ADHD subtypes with and without comorbid IED, ACEs, and internalizing/externalizing problems

For further analyses, we built combined groups according to ADHD severity and IED diagnoses. To guarantee sufficient subsample sizes and to account for the similarities of the very low and low ADHD subtypes as well as the moderate and severe ADHD subtypes, we combined the very low and low subtype to one group called *lowADHD*, and the moderate and severe subtype to one group called *highADHD*, resulting in two equally proportioned ADHD groups (each 50% of all participants). Combined with IED diagnoses, four groups were built: (1) lowADHD^−IED^ (*n* = 67, 42.9%), (2) lowADHD^+IED^ (*n* = 11, 7.1%), (3) highADHD^−IED^ (*n* = 33, 21.2%), and (4) highADHD^+IED^ (*n* = 45, 28.8%). Table 2 presents descriptive results concerning these groups. No differences were found for age, length of detention, years of education, or offenses. Female participants were under-represented in the lowADHD^−IED^ group but over-represented in the highADHD^+IED^ group. The highADHD^+IED^ group showed a significantly higher CTQ-poly score than the lowADHD^−IED^ group. Regarding YSR, participants with clinically significant internalizing problems were over-represented in the highADHD^−IED^ group and under-represented in the lowADHD^−IED^ group. Participants with clinically significant externalizing problems were over-represented in the highADHD^+IED^ group and under-represented in the lowADHD^−IED^ group. Internalizing problem scores of the lowADHD^−IED^ group were significantly lower than scores of the highADHD^−IED^ and highADHD^+IED^ groups, and scores of the lowADHD^+IED^ group were lower than scores of the highADHD^−IED^ group. For externalizing problems, the highADHD^+IED^ group showed the highest scores, followed by those of the highADHD^−IED^ group. Both were significantly higher than scores of the lowADHD^−IED^ group.

**Table 2:** Descriptive differences among ADHD/IED groups

	ADHD/IED group				
	LowADHD^−IED^ (*n* = 67)	LowADHD^+IED^ (*n* = 11)	HighADHD^−IED^ (*n* = 33)	HighADHD^+IED^ (*n* = 45)	
	*n* (AR)	*n* (AR)	*n* (AR)	*n* (AR)	*χ*^2^ (*df*)
Sex					
Male	62 (2.8)	11 (1.6)	24 (− 1.7)	32 (− 2.4)	13.35 (3)**
Female	5 (− 2.8)	0 (− 1.6)	9 (1.7)	13 (2.4)	
Index offenses					
Bodily harm	13 (− 0.6)	3 (0.5)	6 (− 0.6)	12 (0.9)	15.29 (15)
Property offenses	29 (1.7)	3 (− 0.6)	9 (− 1.2)	15 (− 0.4)	
Breach of narcotics law	9 (− 0.9)	4 (1.8)	7 (0.8)	6 (− 0.7)	
Beach of school law/excessive school skipping	7 (− 0.8)	1 (− 0.4)	4 (− 0.1)	8 (1.2)	
Driving without driver’s license	6 (1.5)	0 (− 0.9)	2 (0.1)	1 (− 1.2)	
Others	3 (− 1.1)	0 (− 0.9)	5 (2.0)	3 (− 0.1)	
YSR (categorical)					
Internalizing problems	1 (− 5.0)	1 (− 0.9)	16 (4.6)	13 (1.8)	34.28 (3)***
Externalizing problems	9 (− 4.3)	2 (− 1.0)	13 (1.0)	26 (4.4)	26.13 (3)***

	*M* (SD)	*M* (SD)	*M* (SD)	*M* (SD)	*F* (3, 152)
Age	18.21 (2.01)	18.27 (1.42)	18.88 (2.20)	18.80 (2.35)	1.10
Length of detention (weeks)	2.16 (0.73)	2.09 (0.30)	1.93 (0.50)	2.16 (0.77)	0.91
Years of education	9.38 (0.80)	9.34 (0.67)	9.27 (0.63)	9.13 (0.79)	1.07
CTQ poly score	2.07 (1.28)_a_	2.27 (1.42)_a, b_	2.49 (1.48)_a, b_	2.96 (1.45)_b_	3.70*
YSR (dimensional)					
Internalizing problems	4.06 (4.48)_a_	5.45 (8.99)_a_	16.94 (14.78)_b_	11.69 (9.37)_a,b_	16.32***
Externalizing problems	6.84 (6.98)_a_	9.72 (10.75)_a, b_	14.12 (12.69)_b_	18.29 (12.96)_b_	11.29***

*AR* adjusted residuals. Significant deviations from expected distribution with AR ≤ − 2.0 or AR ≥ 2.0. Groups with the same subscripts (a, b, c, d) did not significantly differ from each other (*p* ≥ 0.05). *p < 0.05, **p ≤ 0.01,***p ≤ 0.001

Results of regression analyses are presented in Tables 3 and 4. Using different models, we found that (1) the CTQ-poly score significantly predicted belonging to the ADHD^+IED^ but not to the ADHD^−IED^ group and (2) high YSR internalizing and externalizing problem scores. Moreover, highADHD^+IED^ and highADHD^−IED^ groups (3) significantly predicted internalizing and externalizing problem scores with stronger effects of the ADHD^−IED^ group on internalizing problems, and the ADHD^+IED^ group on externalizing problems. (4) CTQ-poly score and affiliations to highADHD^+IED^/highADHD^−IED^ groups maintained their predictive effects on internalizing and externalizing problems even when considered simultaneously. Furthermore, predictive effects of sex over and above those of CTQ-poly scores were found as females showed higher risk of belonging to high ADHD groups and of internalizing problems.

**Table 3:** Predictive associations between ACEs and ADHD/IED groups

	LowADHD^+IED^			HighADHD^−IED^			HighADHD^+IED^		
	OR	95% CI	*p*	OR	95% CI	*p*	OR	95% CI	*p*
CTQ poly score	1.10	0.68–1.78	0.697	1.25	0.92–1.70	0.151	**1.61**	**1.19–2.19**	**0.002**
Sex	–	–	–	**0.21**	**0.06–0.69**	**0.010**	**0.20**	**0.06–0.68**	**0.007**

The lowADHD^−IED^ group served as reference group. Sex was coded as female = 1, male = 2. No females were in the lowADHD^+IED^ group. Significant associations in bold

**Table 4:** Predictive associations between ACEs and externalizing/internalizing problems

	YSR internalizing problems				YSR externalizing problems			
	*B*	95% CI	ß	*p*	*B*	95% CI	ß	*p*
Model 1								
CTQ poly score	**1.99**	**0.88–3.10**	**0.27**	**0.001**	**2.46**	**1.24–3.69**	**0.30**	**< 0.001**
Sex	− **5.78**	− **9.93–1.62**	− **0.21**	**0.007**	− 4.41	− 8.98–0.17	− 0.15	0.059
Model 2								
LowADHD^+IED^	1.62	− 4.30–7.54	0.04	0.589	3.02	− 3.78–89.82	0.07	0.381
HighADHD^−IED^	**12.28**	**8.33–16.22**	**0.48**	**< 0.001**	**6.94**	**2.40–11.47**	**0.25**	**0.003**
HighADHD^+IED^	**6.98**	**3.37–10.58**	**0.30**	**< 0.001**	**11.08**	**6.93–15.22**	**0.44**	**< 0.001**
Sex	− 3.05	− 7.07–0.97	− 0.11	0.136	− 1.76	− 6.38–2.86	− 0.06	0.452
Model 3								
CTQ poly score	**1.56**	**0.53–2.60**	**0.21**	**0.003**	**1.77**	**0.58–2.97**	**0.22**	**0.004**
LowADHD^+IED^	1.31	− 4.47–7.09	0.03	0.655	2.67	− 3.97–9.30	0.06	0.429
HighADHD^−IED^	**11.65**	**7.78–15.52**	**0.45**	**< 0.001**	**6.22**	**1.78–10.67**	**0.22**	**0.006**
HighADHD^+IED^	**5.61**	**1.98–9.24**	**0.24**	**0.003**	**9.53**	**5.35–13.70**	**0.38**	**< 0.001**
Sex	− 2.99	− 6.91–0.94	− 0.11	0.135	− 1.69	− 6.20–2.82	− 0.06	0.460

The lowADHD^−IED^ group served as reference group for models 2 and 3. Sex was coded as female = 1, male = 2. Significant associations in bold

## Discussion

This study is the first to explore the occurrence of empirically derived ADHD-subtypes with and without co-existing IED as well as their relations to ACEs and further internalizing and externalizing problems among young offenders. Considering the major relevance of these factors for understanding the occurrence and maintenance of criminal behaviors, our examination of a sample of young offenders at an early stage of criminal development yielded several important findings that expand current knowledge in this field.

Four empirically based ADHD subtypes emerged. They differed on overall severity instead of showing varying symptom patterns, reflecting differences of rather quantitative than qualitative nature. ADHD symptoms were highly prevalent with a quarter of participants reporting at least moderate and one quarter reporting severe ADHD symptomatology. These results are in line with previous findings of high ADHD rates in young and adult offenders and underscore the importance to consider ADHD as a relevant psychiatric disorder in forensic samples [14, 77]. In addition, our results support calls not only to rely on categorical presentations of ADHD but to consider dimensional expressions of ADHD symptomatology/severity [23, 51]. Retz-Junginger and colleagues [51] compared young male detainees with and without ADHD diagnoses on seven of the 10 WR-SB scales and found similar patterns to those found on the subtypes in the present study. Compared to prior studies that have implemented LCA/LPA approaches on ADHD symptomatology [27, 52], we found fewer distinct subtypes, which might be ascribed to the specific composition of our young offender sample in contrast to more general populations as well as the fact that we did not include further co-existing emotional or behavioral symptoms in our LPA. However, the ADHD subtypes found in the present study were more distinguishable than expected, although some differences on further variables of interest were subtle and thus allowed to merge the four subtypes into two groups representing low and high ADHD severity.

A substantial prevalence of DSM-5 oriented IED (36%) was found in the present sample compared to rates reported in general population, psychiatric patient, or other offender samples [34, 35, 57, 78]. This finding indicates that IED is common among young offenders and merits more scientific consideration. However, it is possible that the prevalence found in the present study might be over-estimated at least to some extent due to bias in self-report in contrast to external clinical judgment. Yet, reliance on DSM-5 has been proposed to lead to a higher IED prevalence than earlier considerations of DSM-IV criteria [78]. In addition, young offenders in the present study showed elevated rates of further internalizing and externalizing problems; as found in previous research, externalizing problems reached clinical significance more often than internalizing problems, whereas the latter was still common, highlighting the necessity not to neglect those impairments in offender samples [2].

Our results also underline that the vast majority of young offenders is burdened with ACEs [3, 44]. More than 85% of participants reported at least one of the five assessed ACEs, and more than 28% indicated to have experienced at least four out of five ACE categories. Thus, high rates of ACEs are present in intensive and chronic young offenders [43] but also in those delinquents who are rather at an early stage of criminal development.

Regarding the associations among ADHD, IED, ACEs and further internalizing and externalizing problems, the following results require specific consideration. First, IED was particularly prevalent among participants with severe ADHD. This finding is consistent with research emphasizing the common co-existence of ADHD and IED (and other externalizing disorders) both on behavioral outcomes as well as underlying processes [23, 30, 31]. In their literature review, Gnanavel and colleagues [23] point to similarities in symptomatology (e.g. regarding aggressive and impulsive behavior) und highlight shared genetic as well as environmental risk factors, the latter including disturbed family contexts. Puiu et al. [30] refer to shared associations with emotional lability and irritability as well as comparable expressions of deficient emotion regulation in terms of, e.g., hyperarousal, intrusiveness, or distractibility. Yet, they point out that impulsive aggression of IED subjects appeared to be of greater severity compared to individuals with ADHD or other externalizing disorders. It has been proposed that the intensity of aggression in IED may be traced back to repeated experience of interpersonal ACEs, which contributed to a seriously disturbed development of emotion regulation abilities [46]. Although we are not aware of any previous study that had directly compared differences in the associations of ACEs with ADHD and IED subjects, our findings indicate that an elevated ACE history may increase the risk of IED when co-occurring with elevated ADHD symptomatology. Thus, one could assume that ADHD and IED share etiological and symptomatic factors related to emotional dysregulation, but ACEs intensify problematic outcomes in terms of (severe and impulsive) aggressive behavior. Future research should examine this assumption in more detail. Young offenders high on ADHD with or without IED were, however, not more likely to be detained for violent offenses than other participants. Yet, findings must be interpreted cautiously, because information was available only on the index offenses and not on further/previous crimes. Furthermore, young offenders with severe violent offenses are probably more likely to be found in prison populations than in juvenile detention.

Second, high ADHD severity was related to elevated rates of further internalizing and externalizing problems, highlighting the multiplicity of psychiatric impairments accompanying ADHD [51]. Under consideration of co-existing IED, those participants high on ADHD with IED showed comparably high expressions of externalizing problems, whereas those high on ADHD without IED stood out because of an elevated risk of internalizing problems. Thus, regarding further psychiatric impairments, it appears of major importance not only to consider ADHD but also co-existing IED symptoms. Concerning the debate of the conceptualization of ADHD with and without further emotional and behavioral symptoms [22, 79–81], the co-existence of both disorders in the present sample hints to overlapping features of ADHD (subtypes) and IED-typical presentations of emotional dysregulation, whereas the specific associations with other variables of interest indicate the distinctiveness of both disorders that rather appear to co-exist in the form of (subtype-dependent) comorbidity.

Third, high ADHD severity was related to increased ACE rates. The cumulative ACE score predicted severe ADHD, but only when co-existent with IED. Previous research has yielded different findings concerning the link between ACEs and ADHD. For example, some studies did not find predictive links between ACEs and ADHD [3], whereas others did [40] and others highlighted the role of co-occurring emotional and/or behavioral disturbances in this context [38, 82]. The present findings support the latter and indicate that considering ADHD without the psychosocial background might hinder the detection and development of explanatory approaches with respect to psychiatric burden in young offenders. In addition, cumulated ACEs positively predicted both internalizing and externalizing problems beyond the abovementioned effects of ADHD, pointing to the independent influences of ACEs and ADHD on further psychiatric impairment.

Fourth, the explanatory examination of sex differences indicated that young female offenders were more likely to show severe ADHD with or without IED as well as further behavior problems, whereas no differences were found regarding cumulative ACE burden. These findings are only partially consistent with prior studies. Some researchers reported that ADHD is common in young female detainees [59], whereas others found lower rates of ADHD in young female compared to male offenders [4]. It has been suggested that it requires more severe and frequent criminal behavior for females to get arrested, resulting in an over-representation of severely disturbed young females within forensic samples, most of them with elevated trauma histories [4].

Some qualifications must be considered for the interpretation of the present findings. Due to the primary purpose as pilot/feasibility study, data was solely assessed by self-reports and not expanded to external evaluation (e.g., interviewing) by clinical experts. The validity of self-reports on ADHD symptoms, further psychiatric impairments, and ACEs has been debated, especially in forensic samples [2, 6, 83]. We could not ensure that the young offenders’ educational levels might have affected comprehension of the questionnaires; however, as participants had experiences at least 8 years of school education, we were confident that the majority of young offenders comprehended respective items, and there was also the possibility for participants to ask staff members in case of uncertainties in item comprehension. Additionally, the application of more objective, physiological measures (e.g., electroencephalography (EEG) [31]) would have been interesting but was beyond the scope of the present study. Furthermore, we only assessed five types of ACEs, although previous research on young offenders has yielded interesting findings by including more types of both intra-familial and extra-familial ACEs [53]. We were not able to include data on former crime and re-offending or further biographical information, which are of major importance for the understanding of criminal development. Moreover, the size of the current sample was limited and only contained a small number of female subjects, requiring cautious interpretation and prohibiting generalized implications, particularly regarding sex-differences. A larger sample size would have led to greater statistical power and, thus, to more confidence regarding the interpretation of our results. Eventually, testing multiple hypotheses on one sample has been associated with the risk of type-I error inflation, and the benefits and disadvantages of applying respective correction methods have been subject to scientific debate [84]. For the present study, we decided to consider results with *p*-values below the general *α*-level of 0.05 as statistically significant, yet recommending cautious interpretation and suggesting that conclusions with higher reliability may be drawn from results with *p*-values of and below 0.001. However, we assessed a set of important criminogenic variables in a non-preselected sample of young offenders who were at an early stage of criminal development and who had shown a variety of (rather minor) offenses. These offenders represent a high-risk population for chronic crime as it occurs in real life settings. Thus, our findings are of special importance for developing (1) adequate measures aimed at keeping young offenders from continuing criminal behavior and (2) primary prevention strategies to preclude the occurrence of juvenile criminal conduct in the first place. In addition, we used sophisticated data analysis techniques to derive ADHD subtypes based on empirical rather than theoretical foundation which allowed the “depiction of the way these [young offenders] are naturally sorted” [27]. Furthermore, focusing on the role of IED in forensic samples has been claimed to be of major importance [35].

In conclusion, the present study showed that not only chronic and severe criminals but also young offenders at the beginning of their delinquent development are afflicted with ADHD, IED, ACEs, and further internalizing and externalizing problems. Our findings highlight the need for early identification of crime-promoting factors in young offenders, which requires standardized but individualized assessment of the young offenders’ (or high-risk samples’) risks and needs by psychiatrically/psychologically educated experts. Practitioners in psychiatry, psychology, and law enforcement as well as politicians and other stakeholders need to work together towards the realization and implementation of tailored interventions, not only to protect society from future crime and reduce respective economic costs, but also to allow young offenders and high-risk youths to develop functional and crime-free future prospects.
