# Correction to: ADHD symptom profiles, intermittent explosive disorder, adverse childhood experiences, and internalizing/externalizing problems in young offenders

**DOI:** 10.1007/s00406-021-01308-1

**Published:** 2021-07-20

**Authors:** Steffen Barra, Daniel Turner, Marcus Müller, Priscilla Gregorio Hertz, Petra Retz-Junginger, Oliver Tüscher, Michael Huss, Wolfgang Retz

**Affiliations:** 1 Institute for Forensic Psychology and Psychiatry, Saarland University Hospital, Kirrberger Str. 100, 66421 Homburg, Germany; 2 Department of Psychiatry and Psychotherapy, University Medical Center of the Johannes Gutenberg-University, Mainz, Germany; 3 Department of Child and Adolescent Psychiatry, University Medical Center of the Johannes Gutenberg-University, Mainz, Germany

## Correction to: European Archives of Psychiatry and Clinical Neuroscience 10.1007/s00406-020-01181-4

The article “ADHD symptom profiles, intermittent explosive disorder, adverse childhood experiences, and internalizing/externalizing problems in young offenders”, written by Steffen Barra, Daniel Turner, Marcus Müller, Priscilla Gregorio Hertz, Petra Retz-Junginger, Oliver Tüscher, Michael Huss, and Wolfgang Retz was originally published electronically on the publisher’s internet portal on August 11, 2020 without open access. With the author(s)’ decision to opt for Open Choice the copyright of the article changed to © The Author(s) 2020 and the article is forthwith distributed under a Creative Commons Attribution 4.0 International License, which permits use, sharing, adaptation, distribution and reproduction in any medium or format, as long as you give appropriate credit to the original author(s) and the source, provide a link to the Creative Commons licence, and indicate if changes were made. The images or other third party material in this article are included in the article’s Creative Commons licence, unless indicated otherwise in a credit line to the material. If material is not included in the article’s Creative Commons licence and your intended use is not permitted by statutory regulation or exceeds the permitted use, you will need to obtain permission directly from the copyright holder. To view a copy of this licence, visit http://creativecommons.org/licenses/by/4.0/. Open Access funding enabled and organized by Projekt DEAL.

The original article has been updated.

